# Screening and Characterization of Marine *Bacillus atrophaeus* G4 Protease and Its Application in the Enzymatic Hydrolysis of Sheep (*Ovis aries*) Placenta for the Preparation of Antioxidant Peptides

**DOI:** 10.3390/molecules30102217

**Published:** 2025-05-20

**Authors:** Wei Wang, Guoqing Peng, Jingjing Sun, Chengcheng Jiang, Jianhua Hao, Xiu Zhang

**Affiliations:** 1 Yellow Sea Fisheries Research Institute, Chinese Academy of Fishery Sciences, Qingdao 266071, China; weiwang@ysfri.ac.cn (W.W.); brvu2666@163.com (G.P.); sunjj@ysfri.ac.cn (J.S.); jiangcc@ysfri.ac.cn (C.J.); 2 Jiangsu Collaborative Innovation Center for Exploitation and Utilization of Marine Biological Resource, Lianyungang 222005, China; 3 Ningxia Key Laboratory for the Development and Application of Microbial Resources in Extreme Environments, College of Biological Science and Engineering, North Minzu University, Yinchuan 750021, China; zhangxiu101@aliyun.com

**Keywords:** protease, *Bacillus atrophaeus*, sheep (*Ovis aries*) placenta, characterization, enzymatic hydrolysis, antioxidation

## Abstract

Proteolytic enzymes, which play a crucial role in peptide bond cleavage, are widely applied in various industries. In this study, protease-producing bacteria were isolated and characterized from marine sediments collected from the Yellow Sea, China. Comprehensive screening and 16S rDNA sequencing identified a promising G4 strain as *Bacillus atrophaeus*. Following meticulous optimization of fermentation conditions and medium composition via response surface methodology, protease production using strain G4 was significantly enhanced by 64%, achieving a yield of 3258 U/mL. The G4 protease exhibited optimal activity at 50 °C and pH 7.5, demonstrating moderate thermal stability with 52% residual activity after 30-min incubation at 50 °C—characteristics typical of an alkaline protease. Notably, the enzyme retained over 79% activity across a broad pH range (6–11) and exhibited excellent salt tolerance, maintaining over 50% activity in a saturated NaCl solution. Inhibition by phenylmethylsulfonyl fluoride, a serine protease inhibitor, confirmed its classification as a serine protease. The enzyme’s potential in generating bioactive peptides was further demonstrated through hydrolysis of sheep (*Ovis aries*) placenta, resulting in a hydrolysate with notable antioxidant properties. The hydrolysate exhibited a 64% superoxide anion scavenging activity, surpassing that of reduced glutathione. These findings expand the current understanding of *Bacillus atrophaeus* G4 proteases and provide a foundation for innovative sheep placenta utilization with potential industrial applications.

## 1. Introduction

Proteases, a class of biocatalysts that catalyze the hydrolysis of peptide bonds, play a crucial role in various cellular metabolic pathways and are widely applied across multiple industrial sectors [1]. These enzymes are particularly important in industries such as leather processing, dairy production, diagnostic reagent development, waste management, and precious metal recovery. Notably, they are widely used in detergent formulation, food processing, and pharmaceutical manufacturing [2]. Despite the extensive characterization of protease diversity, only a limited subset exhibits the necessary stability to withstand the harsh conditions common in industrial processes, such as high temperatures, extreme pH, elevated salinity, and exposure to organic solvents [3]. Consequently, there is an urgent need in both applied and fundamental research to identify and characterize novel proteases with exceptional stability and activity under these challenging conditions.

Covering over 71% of the Earth’s surface, the ocean is a vast reservoir of biodiversity, rich in microbial life forms exhibiting a wide range of unique properties [4]. Marine environments are particularly noted for their dense microbial communities, which include extremophilic microorganisms capable of thriving under harsh conditions such as high temperature, salinity, nutrient limitation, and pressure. Such extremophiles have evolved distinct metabolic and physiological traits compared to their terrestrial counterparts [5]. Marine-derived proteases, in particular, exhibit a broader range of catalytic functions and possess distinctive enzymatic properties, including enhanced activity and stability [5]. These properties hold significant potential for driving innovation across diverse sectors, including medical applications [6], the household chemical industry, and food processing [2].

The placenta, a transient yet indispensable organ essential for fetal growth and development, serves as a rich source of essential amino acids, bioactive peptides, vitamins, trace elements, growth factors, and numerous other bioactive compounds [7,8]. Recent studies have shown that enzymatic hydrolysates derived from sheep placenta exhibit a variety of therapeutic properties, including potent antioxidant activity [9] and fatigue-alleviating effects [10]. These bioactivities highlight their potential for a wide range of applications. Despite the abundant availability of sheep placenta resources, especially in China, a large proportion is currently discarded, leading to substantial resource wastage. The Ningxia Tan sheep, a unique Chinese fur breed listed as a national Grade II protected species, has not yet been reported as a source of placenta hydrolysate. The systematic development and strategic utilization of placental materials offer significant practical advantages and considerable market potential, underscoring the need for further research and innovation in this field.

The genus *Bacillus* represents a major source of commercially valuable proteases, noted for their high enzymatic activity, stability across a wide pH and temperature range, and broad substrate specificity. These enzymes also offer advantages such as ease of purification and cost-effectiveness [11,12]. However, empirical studies on proteases derived from *Bacillus atrophaeus* remain limited [13,14]. To optimize the use of marine biological resources and promote the sustainable utilization of sheep placenta, we isolated a *Bacillus atrophaeus* G4 strain from Yellow Sea sediments in China, which produces a protease of notable interest. This protease was subsequently used for the enzymatic hydrolysis of sheep placenta, and the resulting hydrolysates were assessed for their antioxidant potential. The overarching objective of this study is to enhance the value and broaden the application potential of sheep placenta resources.

## 2. Results and Discussion

### 2.1. Strain Screening and Identification

#### 2.1.1. Strain Screening

A total of 493 bacterial strains isolated from Yellow Sea sediments were initially screened using skim milk agar plates to assess proteolytic activity. Among them, 263 strains exhibited proteolytic activity, as evidenced by clear hydrolysis zones surrounding the colonies. Strains forming large hydrolysis zones were selected for further evaluation. Selected strains were cultured in fermentation medium at 30 °C and 180 rpm for 24 h. Following centrifugation at 8000× *g* for 10 min at 4 °C, the supernatant was collected as the crude enzyme extract. Eight strains exhibited protease activity exceeding 100 U/mL. Through successive rounds of fermentation and subculturing, strain G4 was identified as the most promising candidate, exhibiting exceptional protease activity (1759 U/mL) and stable inheritance across passages.

#### 2.1.2. Molecular Identification of Strain G4

**Figure 1:**
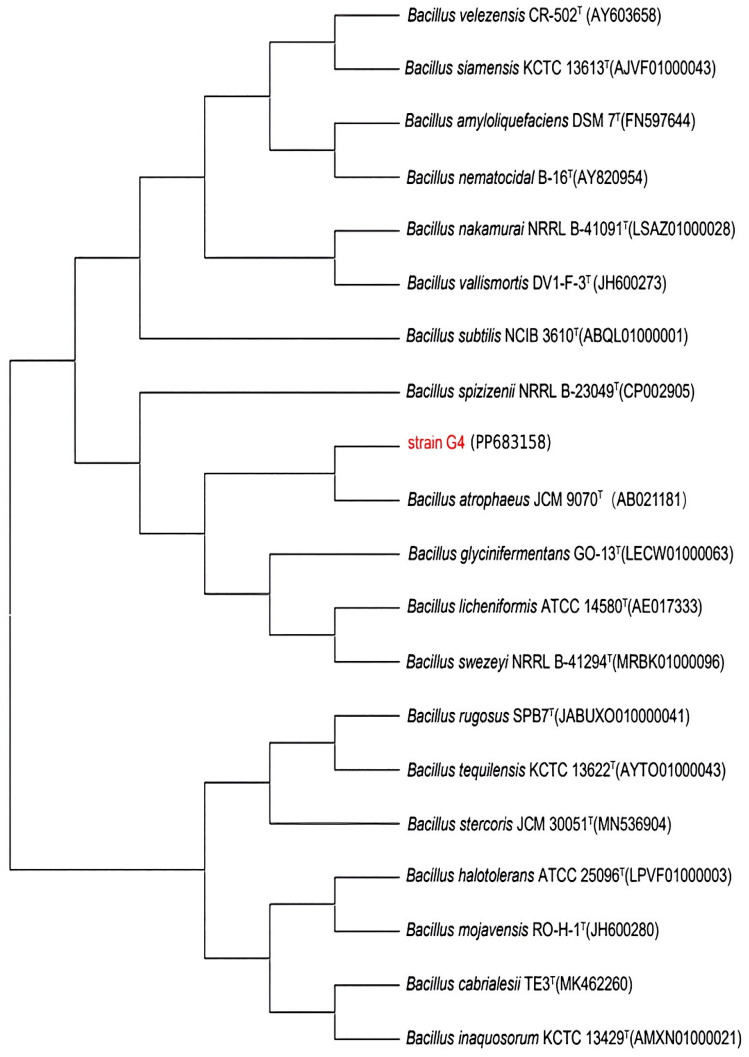
Phylogenetic tree of strain G4 constructed based on 16S rRNA gene sequences, illustrating its phylogenetic placement within the *Bacillus* genus. GenBank accession numbers are shown in parentheses.

The 16S rRNA gene sequence of strain G4 was analyzed using the EzBioCloud database, revealing a 99.9% sequence similarity with *Bacillus atrophaeus* JCM 9070. Sequences of closely related species were selected and aligned using MEGA11.0 for multiple sequence comparison. A phylogenetic tree was then constructed using the neighbor-joining method, as shown in Figure 1.

### 2.2. Optimization of Protease Production by Fermentation of Strain G4

#### 2.2.1. Effect of Cultivation Time

**Figure 2:**
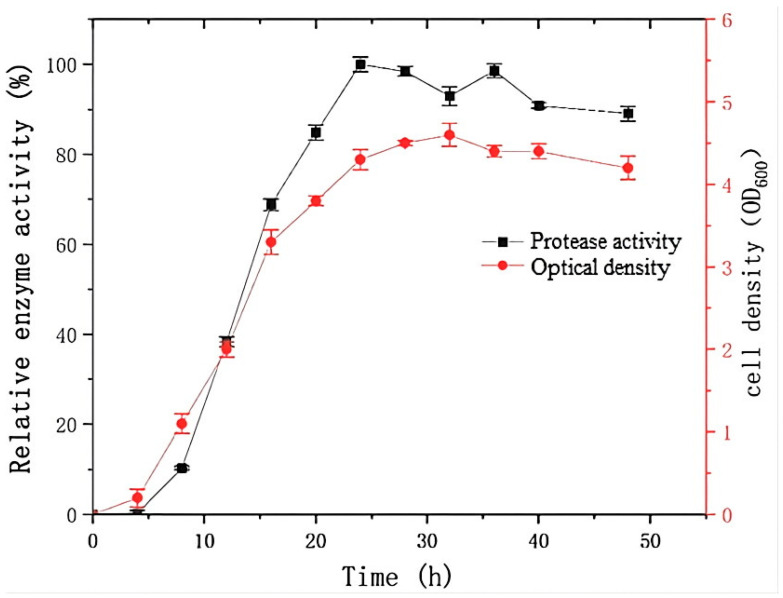
Protease production and growth kinetics of strain G4. The relative activity (100%) corresponds to protease activity of 1700 U/mL.

As shown in Figure 2, a significant increase in the cell density of strain G4 was observed after 4 h of incubation, coinciding with exponential growth in protease production. Maximum protease activity was observed at 24 h. The growth and protease production profile of strain G4 were comparable to that of *Bacillus siamensis* F2, a halotolerant protease-producing strain [15], which reached the stationary phase at 24 h, with no further increase in enzyme yield. However, in contrast to *B. siamensis* F2, which exhibited rapid cell death and a continuous decline in enzyme activity after 24 h of fermentation, *B*. *atrophaeus* G4 showed more stable growth and sustained higher protease activity beyond 24 h.

#### 2.2.2. Effect of Temperature and pH on Protease Production

**Figure 3:**
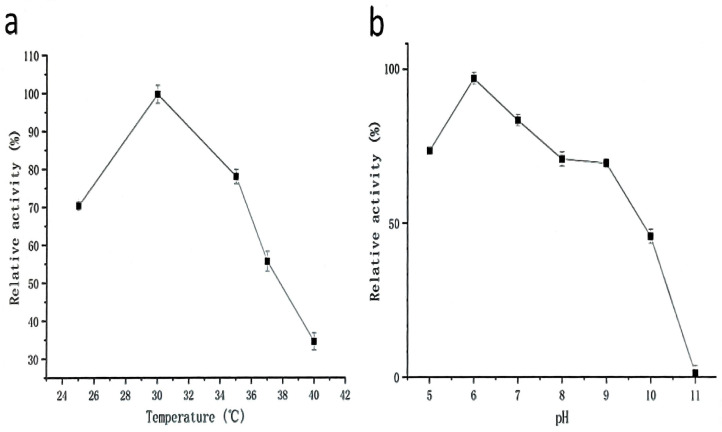
Effect of temperature (**a**) and pH (**b**) on protease production (black squares represent protease activity values) during fermentation by strain G4. The relative activity (100%) corresponds to protease activity of 1700 U/mL. All cultures were grown for 24 h at 200 rpm, with (**a**) pH 6.0 and (**b**) temperature at 30 °C.

Strain G4 exhibited protease production across a temperature range of 25–40 °C, with maximum activity observed at 30 °C (Figure 3a). The effect of pH on protease production during fermentation is shown in Figure 3b. The results indicate that neutral to mildly acidic conditions (pH 6) are most favorable for protease production.

#### 2.2.3. Optimization of the Fermentation Medium for Protease Production

**Figure 4:**
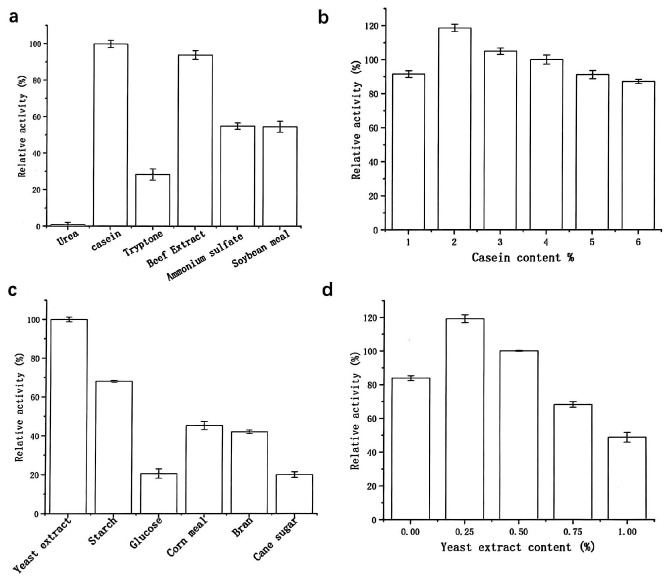
Effects of nitrogen sources (**a**,**b**) and carbon sources (**c**,**d**) on protease production. Nitrogen source screening: The 100% relative activity corresponds to 1700 U/mL (basal medium: 0.5% (*w*/*v*) yeast extract, 1% (*w*/*v*) NaCl); carbon source screening: The 100% relative activity corresponds to 2500 U/mL (basal medium: 2% (*w*/*v*) casein, 1% (*w*/*v*) NaCl).

As shown in Figure 4, preliminary analysis using a one-variable-at-a-time approach revealed that a fermentation medium containing 2% (*w*/*v*) casein, 0.25% (*w*/*v*) yeast extract, and 1% (*w*/*v*) NaCl was optimal for protease production by strain G4. Notably, yeast extract, serving as a carbon source, significantly enhanced protease production compared to other tested sources such as glucose, starch, sucrose, cornmeal, and bran. Even slight variations in the concentration of yeast extract led to substantial changes in protease yield. This enhancement is attributed to the rich nutritional composition of yeast extract, which contains amino acids, essential vitamins, and carbohydrates that support cell growth and enhance the metabolic activity of the bacterium [16].

#### 2.2.4. Optimization of Enzyme Production Using Response Surface Methodology

In a one-factor-at-a-time experiment, a single variable is varied while all others are kept constant. While this method offers preliminary insights and allows quantification of individual factor effects, it is limited in evaluating interactions among variables and identifying optimal parameter combinations. To better understand the interactions among variables and determine the optimal response region, response surface methodology was employed.

Based on the results of single-factor experiments, a central composite design was used to optimize the fermentation conditions. Using Design-Expert 10.0 software, the concentrations (*w*/*v*) of casein, yeast extract, and NaCl were selected as key influencing factors and defined as independent variables (Table 1).

The experimental data for protease activity (R) were fitted using a quadratic multiple regression model in Design-Expert software. The resulting equation, expressing protease activity (R) as a function of casein content (A), yeast extract content (B), and NaCl concentration (C), is presented below:

R = 3140.62 + 122.24A + 602.78B + 42.78C − 134.53AB + 117.39AC − 44.15BC − 498.95A^2^ − 717.51B^2^ − 207.29C^2^

Analysis of variance and significance testing of the quadratic model were also performed using Design-Expert. The results are summarized in Table 2. The model’s *p*-value (<0.0001) indicates a highly significant fit. Among the linear terms, both A (casein content) and B (yeast extract content) had significant effects on protease production, demonstrating their substantial influence within the tested ranges (casein: 1–3% (*w*/*v*); yeast extract: 0–0.5% (*w*/*v*)). In contrast, C (NaCl concentration) exhibited a *p*-value > 0.05, indicating no statistically significant effect on enzyme production within the range of 0.5–1.5% (*w*/*v*).

The relative influence of the three factors on enzyme production followed the order: B > A > C. The lack-of-fit *p*-value (0.2296 > 0.05) was not significant, supporting the adequacy of the model. The coefficient of determination (R^2^ = 0.9830) and the adjusted coefficient of determination (R^2^adj = 0.9611) were both close to 1, indicating a strong correlation between predicted and actual values. Additionally, the predicted R-Squared (R^2^pred = 0.8201) was in good agreement with R^2^adj, with a difference of less than 0.2, confirming the model’s robustness and predictive accuracy.

In the regression equation, the negative coefficients of the quadratic terms (A^2^, B^2^, C^2^) indicate a downward-opening response surface, suggesting the presence of a maximum point. Among the primary terms, B was identified as significant, while A was highly significant. In the quadratic terms, A^2^, B^2^, and C^2^ all exhibited high significance, further supporting the model’s reliability in describing the system.

To validate the accuracy of the response surface methodology, a verification experiment was conducted under the predicted optimal conditions: 2.08% (*w*/*v*) casein, 0.36% (*w*/*v*) yeast extract, and 1.04% (*w*/*v*) NaCl, with a predicted protease activity of 3271 U/mL. To accommodate practical experimental constraints, the conditions were adjusted to 2.1% (*w*/*v*) casein, 0.36% (*w*/*v*) yeast extract, and 1% (*w*/*v*) NaCl. Three replicate experiments were conducted under these conditions, yielding a measured protease activity of 3258 U/mL. This close agreement between the predicted and experimental values confirms the validity and reliability of the response surface model. The optimized conditions enhanced protease production by 64% compared to the baseline culture system.

### 2.3. Characterization of the Purified Protease

**Figure 5:**
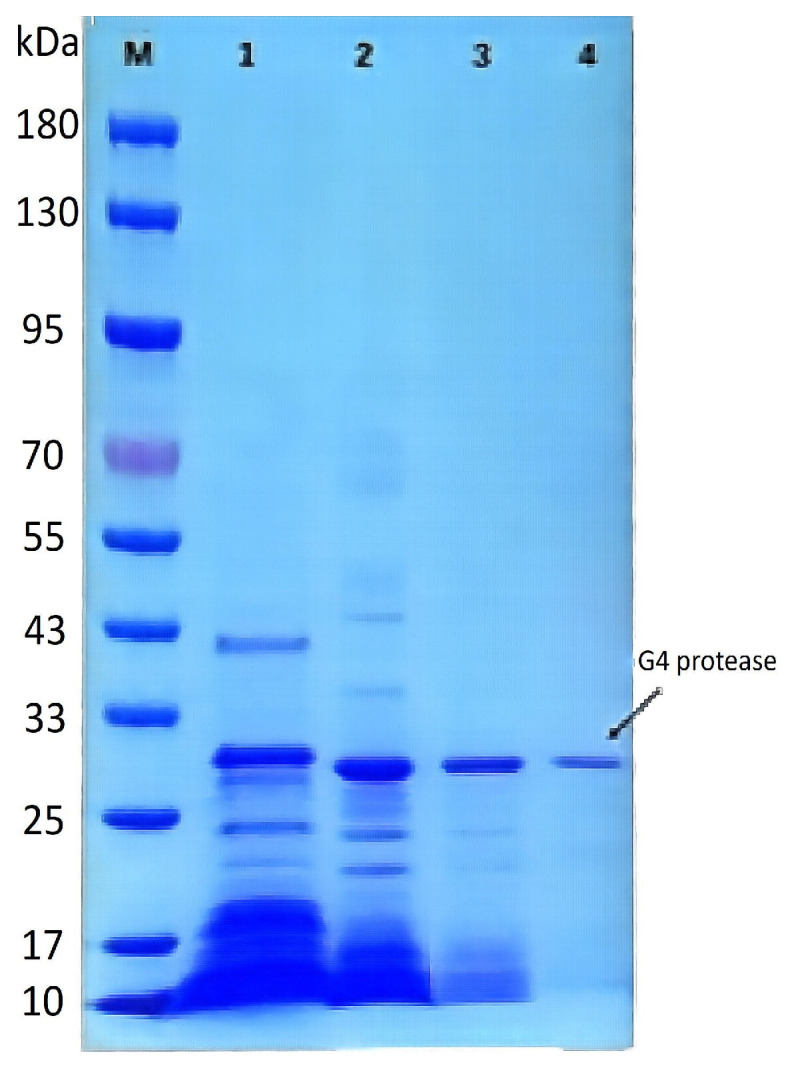
SDS-PAGE analysis of the purification process of protease from strain G4. M: Pre-stained protein molecular weight marker; Lane 1: Crude extract of protease from strain G4; Lane 2: Protease fraction after 20–60% (NH_4_)_2_SO_4_ precipitation; Lane 3: Protease fraction after DEAE FF ion-exchange chromatography; Lane 4: Final purified protease.

Purification of the protease derived from strain G4 resulted in a 25-fold increase in purity, with a specific activity of 10,976 U/mg and a final yield of 4% (Table 3). The molecular weight of the purified protease was estimated to be approximately 29 kDa, as determined by SDS–PAGE (Figure 5). The protease derived from *B. atrophaeus* strain 08 exhibited minimal caseinolytic activity, demonstrating a striking contrast to the high activity observed in the G4 protease [13].

#### 2.3.1. Effect of Temperature on the Activity and Stability of Protease from Strain G4

**Figure 6:**
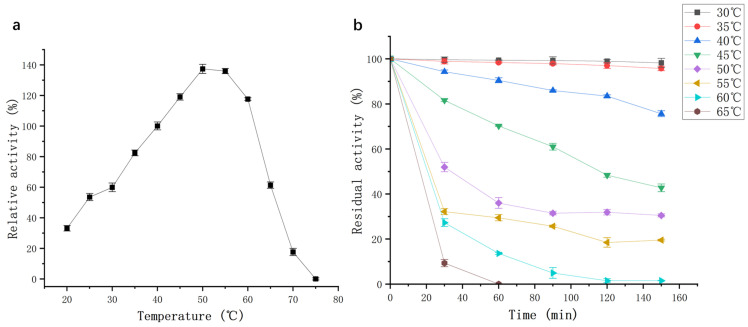
(**a**) Effect of temperature on protease activity (black squares); (**b**) Effect of temperature on thermal stability of protease activity.

Temperature is a key parameter affecting enzymatic activity. As shown in Figure 6a, the purified protease from strain G4 exhibited activity across a broad temperature range, retaining over 40% of its activity between 25 °C and 65 °C, with an optimal temperature of 50 °C. These findings are consistent with previous reports on proteases from *B. siamensis* F2 [15] and *B. subtilis* [17], which also exhibited maximum activity at 50–60 °C. The high relative activity at elevated temperatures suggests that the G4 protease has strong potential for industrial applications operating under medium to high temperature conditions.

Thermal stability assays showed that the enzyme maintained its activity with minimal loss when incubated at 30–35 °C for 150 min (Figure 6b). At 40 °C, 80% of the initial enzyme activity was retained after 150 min. However, when incubated at 50–55 °C, enzyme activity declined sharply within the first 30 min but subsequently stabilized, with 20% residual activity remaining at 55 °C after 150 min. However, the G4 protease was not a thermophilic enzyme, exhibiting lower optimal temperature and thermostability compared to protease SAPNIJ derived from the thermophilic strain *B. atrophaeus* NIJ [14]. Considering the G4 protease’s optimal temperature, thermal stability, and industrial applicability, a working temperature range of 20–60 °C is recommended for practical use.

#### 2.3.2. Effects of pH on Activity and Stability of Protease from Strain G4

**Figure 7:**
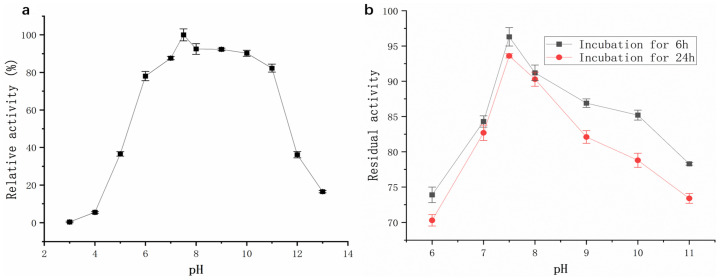
(**a**) Effect of pH on protease activity (black squares); (**b**) Effect of pH on the pH stability of protease activity.

The enzyme exhibited high proteolytic activity across a broad pH range of 6–11 (Figure 7a). Relative activity remained above 78%, with maximum activity observed at pH 7.5. As shown in Figure 7b, the protease from strain G4 exhibited minimal activity loss after incubation at various pH levels for 6 h and 24 h. At pH 7.5, enzyme activity decreased by only 3.8% after 6 h and 6.6% after 24 h of incubation. Furthermore, under the strongly alkaline condition (pH 11), the enzyme retained 73.4% of its initial activity after 24 h (Figure 7b). These results indicate that the enzyme exhibits remarkable stability under both neutral and alkaline pH conditions.

The pH stability of the G4 protease was markedly superior to that of the protease from *B. velezensis* Z-1 reported by Lu et al. [18] as well as a protease from *Bacillus* sp. [19]. This robust pH tolerance makes the G4 protease a promising candidate for use in detergent formulations, as laundry detergents typically function within a pH range of 7–11 [20].

#### 2.3.3. Effect of NaCl on the Activity and Salt Stability of the Protease

**Figure 8:**
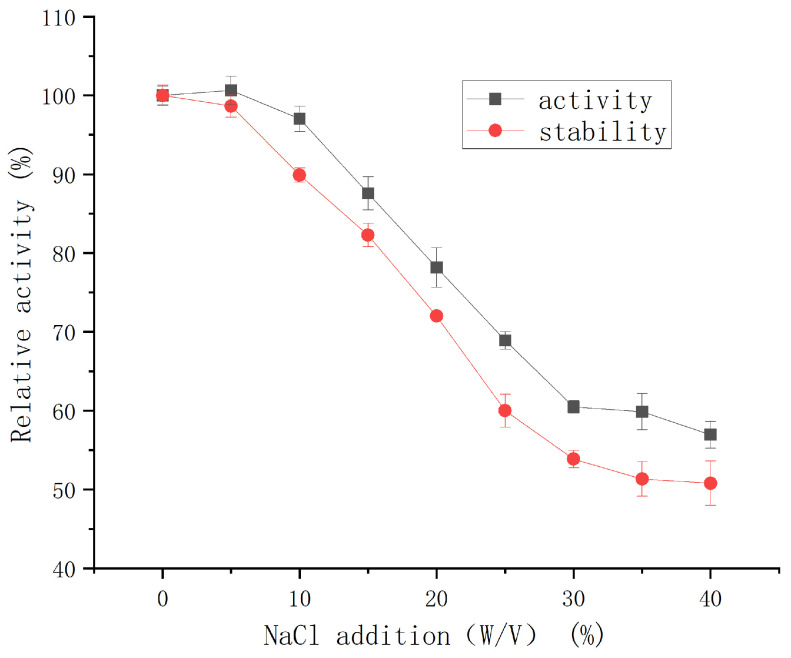
Effect of NaCl concentration on the activity and salt stability of protease from strain G4.

The protease retained >97% of its initial activity across a broad NaCl concentration range (0–10% (*w*/*v*)) (Figure 8). As the NaCl concentration increased, enzymatic activity gradually declined; nevertheless, the enzyme still maintained 57% relative activity in a 40% (*w*/*v*) NaCl-saturated solution (5.4 M). Short-term stability assays (24 h, 4 °C) further confirmed negligible activity loss at ≤5% (*w*/*v*) NaCl. These results indicate that the protease exhibits remarkable stability under high-salt conditions.

This level of salt tolerance is superior to that of a halotolerant metalloprotease from *Vibrio* sp. LA-05 [21], which retained less than 20% of its initial activity under comparable conditions. G4 protease also exhibited better salt tolerance (50% activity retention in 5.4 M NaCl, 4 °C for 24 h) than halotolerant protease SC-lasB (30% retention in 3 M NaCl) [22]. Salt-tolerant proteases (STPs) are considered highly promising for industrial applications due to their superior salt resistance, broader adaptability, and efficient catalytic performance under extreme conditions compared to conventional proteases. In the food processing industry, STPs are especially valuable in the production of high-salt foods such as soy sauce, cheese, shrimp paste, and sausages [23,24]. Given its exceptional salt tolerance, the G4 protease exhibits strong potential for application in high-salt food processing.

#### 2.3.4. Effect of Metal Ions on the Activity of Protease from Strain G4

As shown in Table 4, Mn^2+^ exhibited a slight activating effect on the activity of the protease. In contrast, Fe^3+^ and Al^3+^ completely inhibited protease activity at 10 mM. Similarly, Cu^2+^ and Zn^2+^ significantly suppressed protease activity. G4 protease is inhibited by Zn^2+^, Ni^2+^, Fe^3+^, and Al^3+^, consistent with reports of other proteases (e.g., Zn^2+^ inhibition [14,18,21]; Ni^2+^ [14,18,21]; Fe^3+^ [1]; Al^3+^ [21]). In contrast, Mn^2+^ exhibits divergent effects across proteases: it activates G4 (this study), SAPNIJ [14], strain Z-1 protease [18], and *Nocardiopsis xinjiangensis* protease [3], while demonstrating inhibitory effects on some *Bacillus* proteases [1,11,17], and additional reported cases [12,21]. This dual regulatory role of Mn^2+^—acting as either an activator or inhibitor depending on the protease—warrants further investigation to elucidate the underlying mechanisms.

#### 2.3.5. Effect of Inhibitors, Denaturants, and Surfactants on the Activity of the G4 Protease

The effects of various inhibitors, denaturants, and surfactants on the activity of the G4 protease are summarized in Table 5. Phenylmethylsulfonyl fluoride (PMSF), a widely used inhibitor of serine proteases, significantly suppressed the activity of the G4 protease. At 1 mM, PMSF inhibited 80% of protease activity, whereas at 10 mM, the enzyme was almost completely inactivated. This concentration-dependent inhibition aligns with numerous reports of serine proteases being fully inactivated by 1–5 mM PMSF in the literature [11,12,14,23,25,26]. These results suggest the involvement of serine residues at the active site, confirming that the G4 protease is a serine-type enzyme.

Similar to the proteases from *Bacillus mojavensis* (BM1/BM2) [11] and marine *Vibrio* sp. LA-05 [21], the activity of G4 protease was inhibited by EDTA in a concentration-dependent manner (Table 5). Treatment with EDTA at 1 mM and 10 mM resulted in residual activities of 62.5% and 48.9%, respectively. In contrast, 1,10-phenanthroline, another metal-chelating agent, had negligible effect (<5% inhibition) on the activity of the G4 protease, matching typical serine protease behavior [23,25]. These results suggest that specific metal ions may be involved in maintaining the active conformation of the enzyme. Dithiothreitol treatment (1–10 mM) inhibited G4 protease activity by less than 10%, mirroring the dithiothreitol-insensitive nature characteristic of serine-type proteases [14].

Common nonionic surfactants, such as Tween 80 and OP-10, showed no significant effect on the activity of the G4 protease. This observation aligns with the findings of Suwannaphan et al. [25], who reported that protease activity remained largely unaffected in the presence of 0.5% and 1.0% Tween 20, Tween 80, and Triton X-100, with over 80% activity retained.

### 2.4. Antioxidant Activity of Sheep Placenta Hydrolysates

#### 2.4.1. Effect of Hydrolysis Time on the Antioxidant Activity of Sheep Placenta Hydrolysates

**Figure 9:**
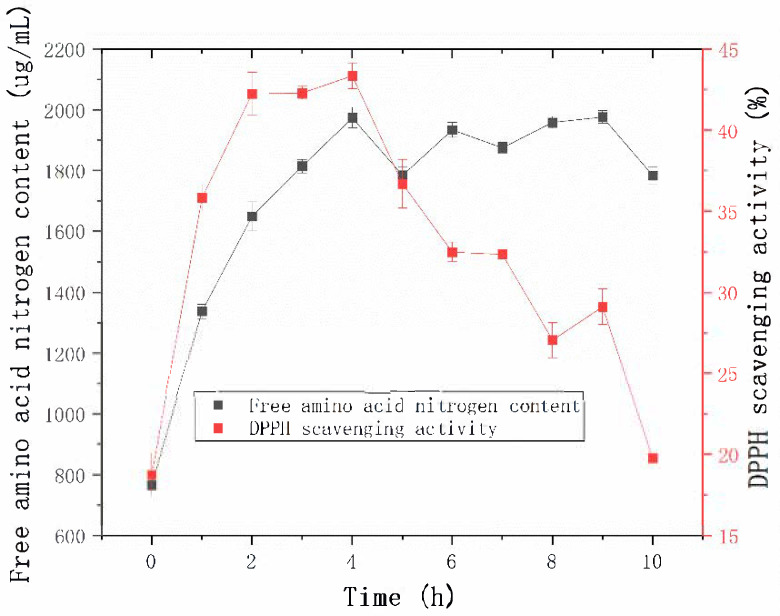
Effect of hydrolysis time on free amino acid content and DPPH radical scavenging activity.

The productivity curve is a valuable tool for monitoring product yield over time under specific conditions, thereby enabling optimization and cost reduction [27]. As shown in Figure 9, the free amino acid content in the enzymatically hydrolyzed sheep placenta reached its maximum at 4 h. The enzymatic efficiency observed in this study exceeded that of the protease from *B. velezensis* Z-1, as reported by Lu et al. [18].

The 2,2-Diphenyl-1-picrylhydrazyl (DPPH) radical scavenging activity was used as a marker of antioxidant capacity to assess the effect of hydrolysis time on the antioxidant properties of the hydrolysates [28]. The DPPH radical scavenging activity increased steadily during the first 2 h of hydrolysis and stabilized between 2 h and 4 h. At 4 h, the DPPH radical scavenging activity reached its maximum value. These results suggest that prolonged hydrolysis may lead to the degradation or inactivation of antioxidant peptides, thereby reducing the overall antioxidant activity of the hydrolysates [29].

#### 2.4.2. Evaluation of the Antioxidant Activity of the Hydrolysate

**Figure 10:**
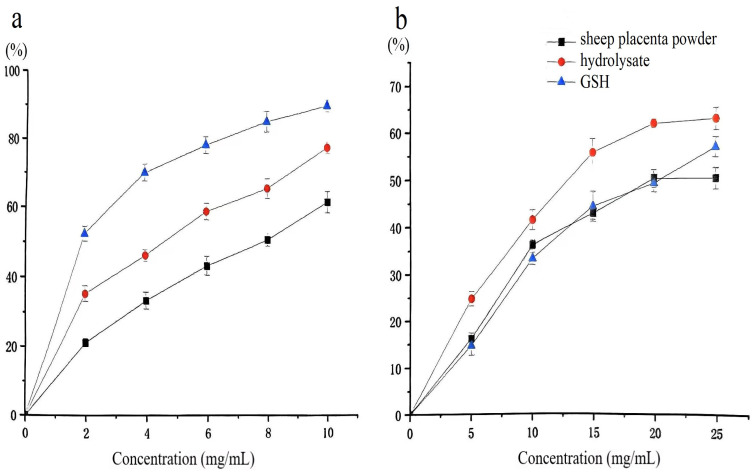
(**a**) DPPH radical scavenging activity of the hydrolysate; (**b**) Superoxide anion radical scavenging activity of the hydrolysate.

DPPH is a stable free radical widely used to evaluate the antioxidant capacity of various food-related substances due to its chemical stability and cost-effectiveness [30]. In this assay, antioxidant compounds mediate radical scavenging through hydrogen atom transfer or single electron transfer mechanisms, with the reaction progress monitored spectrophotometrically at 517 nm following 30 min of incubation under standardized conditions. Glutathione (GSH), a well-known reducing peptide, has been extensively studied for its potent antioxidant properties [31,32]. As shown in Figure 10a, the DPPH radical scavenging activity of the sheep placenta hydrolysate increased with increasing concentration. At 10 mg/mL, the DPPH radical scavenging activity reached 78.3%, showing a 15.7% improvement over the non-hydrolyzed sample and only 12% lower than that of GSH. These results suggest that enzymatic hydrolysis significantly enhances the DPPH scavenging activity of sheep placenta, yielding antioxidant effects comparable to GSH.

Hydrolysis of sheep placenta using papain (4000 U/g) and extraction by-products (10 mg/mL) yielded peptides with a DPPH radical scavenging activity of up to 87% [33]. In this study, untreated sheep placenta was used as the raw material. Although the resulting hydrolysate exhibited a slightly lower DPPH scavenging activity (78.3%), the enzyme dosage (1600 U/g) was more economical.

Superoxide anions, generated during biological metabolic processes, can cause crosslinking or degradation of macromolecules such as proteins, lipids, and nucleic acids. This oxidative damage leads to loss of structural integrity and biological function, accelerates aging, and contributes to the development of various diseases [34]. As shown in Figure 10b, the sheep placenta hydrolysate produced using protease from strain G4 exhibited superior superoxide radical scavenging activity compared to GSH. At 25 mg/mL, the superoxide radical scavenging activity of the hydrolysate reached 64.1%, which was 5.9% higher than that of GSH.

Although bioactive peptides can be directly extracted from sheep placenta [8,35], antioxidant peptides from this source are typically obtained through enzymatic hydrolysis. Previous studies have reported the preparation of sheep placenta antioxidant peptides using dual proteases [9,10] or ultrasound-assisted enzymatic digestion [10]. However, this study demonstrates that comparable antioxidant results can be achieved using a single G4 protease, offering a simpler and more efficient approach.

## 3. Materials and Methods

### 3.1. Screening of Protease-Producing Strains

Primary Screening: The screening medium consisted of 2% (*w*/*v*) skim milk, 0.25% (*w*/*v*) yeast extract, 2% (*w*/*v*) agar, and 1% (*w*/*v*) NaCl. Wells were created in the solidified medium using a sterile hole punch. A total of 493 bacterial isolates, previously activated from Yellow Sea sediment samples, were inoculated by adding 50 μL of each bacterial suspension into individual wells. The inoculated plates were incubated at 30 °C for 24 h. Isolates exhibiting clear zones of proteolysis, indicated by transparent halos in the medium, were selected for further purification and cryopreservation.

Re-screening Process: Isolates exhibiting significant proteolytic activity in the primary screening were subcultured in seed medium composed of 1% (*w*/*v*) tryptone, 0.5% (*w*/*v*) yeast extract, and 1% (*w*/*v*) NaCl, followed by incubation at 30 °C for 24 h. Subsequently, 2% (*v*/*v*) of each culture was inoculated into fermentation medium (4% (*w*/*v*) casein, 0.5% (*w*/*v*) yeast extract, and 1% (*w*/*v*) NaCl) for protease production. Fermentation was carried out in a shaking incubator (Zhichu, Shanghai, China) at 30 °C and 180 rpm for 24 h. Following centrifugation (10,000× *g*, 10 min, 4 °C), the supernatant was collected for protease activity assay. The isolate exhibiting the highest enzymatic activity, designated as strain G4, was selected for further characterization.

### 3.2. Protease Activity Assay

Protease activity was quantified using a modified version of the method described by Lowry et al. [36]. Briefly, 200 μL of enzyme solution (600 U) was mixed with an equal volume of 10 g/L casein solution and incubated at 40 °C for 10 min. The reaction was terminated by the addition of 400 μL of 400 mM trichloroacetic acid. Then, 100 μL of the reaction supernatant was mixed with 500 μL of 400 mM Na_2_CO_3_ and 100 μL of Folin–Ciocalteu reagent. After 20 min of incubation at room temperature, absorbance was measured at 680 nm. One unit of enzyme activity (U) was defined as the amount of enzyme that catalyzes the release of 1 µg of tyrosine-equivalent amino acids per minute under the specified experimental conditions.

### 3.3. Molecular Identification of Bacillus atrophaeus G4

Genomic DNA from strain G4 was extracted using a commercial bacterial DNA extraction kit (Tiangen, Beijing, China). The 16S rRNA gene was amplified via PCR using universal primers 27F (5′-AGAGTTTGATCCTGGCTCAG-3′) and 1492R (5′-ACGGGCGGTGTGTACAAG-3′). The amplified 16S rRNA gene sequence (GenBank accession number: PP683158) was analyzed using the EzBioCloud database for sequence homology comparison [37]. Nineteen closely related strains with the highest sequence similarity were selected for phylogenetic analysis. Phylogenetic relationships were inferred using the neighbor-joining method with 1000 bootstrap replicates in MEGA 11.0 software [38], thereby determining the taxonomic position of strain G4 within the genus *Bacillus*.

### 3.4. Optimization of Protease Production by Bacillus atrophaeus G4 Fermentation

Initial fermentation conditions were established using a basal medium containing 4% (*w*/*v*) casein, 0.5% (*w*/*v*) yeast extract, and 1% (*w*/*v*) NaCl, with incubation at 30 °C and pH 7.0 for 24 h.

A one-factor-at-a-time approach was employed to optimize fermentation parameters, including temperature, pH, incubation time, NaCl concentration, and various carbon and nitrogen sources. Following fermentation, cultures were centrifuged (8000× *g*, 10 min, 4 °C), and protease activity in the resulting supernatant was measured using the standard assay.

Based on the results of single-factor experiments, a Box–Behnken design was implemented using Design-Expert 10.0 software, following the response surface methodology described by Hassabo [39]. Three independent variables—casein content, yeast extract concentration, and NaCl concentration—were optimized, with protease activity as the response variable. Experimental data were analyzed through response surface analysis and model validation to determine the optimal fermentation conditions.

### 3.5. Purification of Protease from Strain G4

Protease purification was carried out using a three-step procedure comprising ammonium sulfate fractionation, ion-exchange chromatography, and ultrafiltration [40]. The fermentation broth was centrifuged (8000× *g*, 10 min, 4 °C) to remove cells. The resulting supernatant was sequentially precipitated with ammonium sulfate at 20% and 60% saturation. The 60% saturation precipitate was dissolved in 20 mM phosphate buffer (pH 7.5) and dialyzed overnight at 4 °C.

The dialyzed sample was loaded onto a HiTrap™ DEAE FF anion-exchange column (Cytiva, Uppsala, Sweden) pre-equilibrated with 20 mM phosphate buffer (pH 7.5). Bound proteins were eluted with a linear NaCl gradient (0–1 M) at a flow rate of 3 mL/min. Fractions exhibiting protease activity were pooled and concentrated using an Amicon^®^ Ultra-15 (Merck, Billerica, MA, USA) centrifugal filter (30 kDa cutoff, 5000× *g*, 15 min, 4 °C). Protein concentration and protease activity were monitored at each step of the purification process [26].

### 3.6. Protein Analysis

Protein concentration was measured using a BCA Protein Assay Kit (Solarbio, Beijing, China), following the manufacturer’s instructions. The purity and molecular weight of the purified protease were analyzed by sodium dodecyl sulfate-polyacrylamide gel electrophoresis (SDS-PAGE) using a 4–20% precast gradient gel (Tsingke, Beijing, China) [41]. Before electrophoresis, samples were mixed with 5× loading buffer containing 5% (*v*/*v*) 2-mercaptoethanol and heated at 95 °C for 5 min to ensure complete protein denaturation. Electrophoresis was carried out at a constant voltage of 120 V until the dye front reached the bottom of the gel. Protein bands were visualized using Coomassie Brilliant Blue R-250 staining.

### 3.7. Enzymatic Characterization of G4 Protease

Temperature and pH Optima: Optimal temperature and pH values for protease activity were determined by incubating the purified enzyme at temperatures ranging from 20 °C to 75 °C (in 5 °C intervals) and pH values from 3.0 to 13.0.

Thermal and pH Stability: Thermal stability was evaluated by incubating the enzyme at temperatures ranging from 30 °C to 65 °C (in 5 °C intervals) for 30–150 min. pH stability was assessed by incubating the enzyme at pH 6.0–11.0 for 1–24 h. Residual activity was measured after each treatment to establish the enzyme’s stability profile.

Metal Ion Effects: The influence of various metal ions (Cu^2+^, Fe^3+^, Mg^2+^, K^+^, Zn^2+^, Ni^2+^, Co^2+^, Mn^2+^, Al^3+^, and Ca^2+^) on protease activity was assessed at final concentrations of 1 mM and 10 mM. Enzyme activity was measured relative to a control without metal ions.

NaCl Tolerance: The effect of NaCl on protease activity was evaluated at 40 °C and pH 7.5, using NaCl concentrations ranging from 0% to 40% (*w*/*v*). Salt tolerance was assessed by measuring residual activity after 24 h of incubation with NaCl.

Inhibitor and Surfactant Effects: The impact of various compounds—including protease inhibitors, reducing agents, and surfactants—on protease activity was investigated. Each compound was tested at appropriate concentrations to evaluate its individual effect on enzymatic activity.

### 3.8. Preparation of Sheep Placenta Hydrolysates Using G4 Protease

Fifty grams of fresh Ningxia Tan sheep placenta powder (Yibaisheng, Yinchuan, China) was homogenized in 50 mM phosphate buffer (pH 7.5) on ice for 20 min. The homogenate was enzymatically digested with G4 protease (1600 U/g substrate) at 40 °C for 4 h with continuous shaking at 150 rpm. The reaction was terminated by boiling for 10 min, followed by centrifugation at 8000× *g* for 15 min at 4 °C. The resulting supernatant was collected and freeze-dried to obtain powdered hydrolysates.

Polypeptide content was quantified using Biuret test reagents (Yuanye, Shanghai, China). The concentration of free amino acid nitrogen was determined using the ninhydrin method [42]. Briefly, 100 μL of ninhydrin reagent (containing 0.5% ninhydrin, 0.3% fructose, 10% Na_2_HPO_4_·10H_2_O, and 6% KH_2_PO_4_) was mixed with 200 μL of hydrolysate solution. After heating at 100 °C for 15 min and cooling to room temperature, 500 μL of 40% ethanol was added. Absorbance was measured at 570 nm using appropriate standard curves for quantification.

### 3.9. Evaluation of Antioxidant Activity of G4 Protease Hydrolysates

Preparation of Peptide Solutions: Lyophilized sheep placenta hydrolysates were dissolved in deionized water to obtain peptide solutions at concentrations of 2.0, 4.0, 6.0, 8.0, and 10.0 mg/mL, based on their measured polypeptide content.

DPPH Radical Scavenging Activity: Antioxidant activity was assessed using a DPPH assay kit (Solarbio, Beijing, China). Briefly, 50 μL of each peptide solution was mixed with 200 μL of DPPH working solution and incubated in the dark at room temperature for 30 min. Absorbance was measured at 517 nm using a spectrophotometer. Glutathione (1 mg/mL) served as the positive control. The scavenging activity was calculated using the following equation:

Scavenging activity (%) = (1 − A_sample_/A_control_) × 100

where A_sample_ and A_control_ represent the absorbance of the sample and control, respectively.

Superoxide Anion Scavenging Activity: Superoxide anion radical scavenging activity was measured using a commercial assay kit (Epsilon, Beijing, China), following the manufacturer’s instructions. The reaction mixture was incubated at 37 °C for 40 min, and absorbance was measured at 550 nm.

## 4. Conclusions

In this study, a high-yield protease-producing strain, G4, was isolated from sediments of the Yellow Sea. Under optimized fermentation conditions, the protease activity reached 3258 U/mL. The protease exhibited high activity and stability across a temperature range of 25–55 °C and a pH range of 6.0–11.0, with notable retention of 20% activity after 2.5 h at 55 °C and 78% activity following 6 h incubation at pH 11.0. Comparative analysis revealed superior thermal resistance to papain (90% vs. 75% residual activity at 40 °C 1 h) [43]. These properties confirm its suitability for industrial applications under moderate temperatures and neutral to alkaline conditions. Additionally, the protease demonstrated remarkable salt tolerance, suggesting its applicability in high-salt food processing.

The enzymatic hydrolysis of sheep placenta using the G4 protease was successfully conducted, yielding hydrolysates with significantly enhanced scavenging activity against both DPPH and superoxide radicals. Notably, the superoxide radical scavenging activity of the hydrolysate surpassed that of GSH, highlighting the potential of G4 protease in the production of bioactive peptides.

While these findings demonstrate the promising applications of G4 protease, its potential likely extends beyond the current scope of investigation. Further research is warranted to explore its commercial applications and optimize its use in various industries.

## Figures and Tables

**Table 1:** Box-Behnken design matrix and experimental results.

Run	A: Casein Content (%)	B: Yeast Extract Content (%)	C: NaCl Addition (%)	R: Protease Activity (U/mL)
1	1	0	1	1191
2	3	0	1	1527
3	1	0.5	1	2590
4	3	0.5	1	2388
5	1	0.25	0.5	2294
6	3	0.25	0.5	2480
7	1	0.25	1.5	2154
8	3	0.25	1.5	2810
9	2	0	0.5	1493
10	2	0.5	0.5	2862
11	2	0	1.5	1658
12	2	0.5	1.5	2850
13	2	0.25	1	3144
14	2	0.25	1	2998
15	2	0.25	1	3195
16	2	0.25	1	3090
17	2	0.25	1	3278

**Table 2:** Analysis of variance for the protease activity regression model.

Source	Sum of Squares	df	Mean Square	F-Value	*p*-Value
Model	6.880 × 10^6^	9	7.645 × 10^5^	44.89	<0.0001
A-casein	1.195 × 10^5^	1	1.195 × 10^5^	7.02	0.0330
B-Yeast extra	2.907 × 10^6^	1	2.907 × 10^6^	170.70	<0.0001
C-NaCl	14,644	1	14,644	0.86	0.3846
AB	72,395	1	72,395	4.25	0.0781
AC	55,117	1	55,117	3.24	0.1150
BC	7796	1	7796	0.46	0.5204
A^2^	1.048 × 10^6^	1	1.048 × 10^6^	61.56	0.0001
B^2^	2.168 × 10^6^	1	2.168 × 10^6^	127.30	<0.0001
C^2^	1.809 × 10^5^	1	1.809 × 10^5^	10.62	0.0139
Residual	1.192 × 10^5^	7	17,028		
Lack of Fit	74,324	3	24,775	2.21	0.2296
Pure Error	44,875	4	11,219		
Cor Total	6.999 × 10^6^	16			
C.V.% = 5.28 R^2^ = 0.9830 R^2^adj = 0.9611 R^2^pred = 0.8201					

**Table 3:** Summary of the purification of crude protease from strain G4.

Purification Step	Total Protein (mg)	Total Activity (U)	Specific Activity (U/mg)	Purification Fold	Recovery (%)
Crude	305.30	133,790	438	1.00	100.00%
20–60% (NH_4_)_2_SO_4_	20.38	94,496	4637	10.58	70.63%
DEAE FF	9.30	69,424	7467	17.04	51.89%
Ultra-15	0.50	5445	10,976	25.05	4.07%

**Table 4:** Effect of metal ions on the activity of protease from strain G4.

Metal Ion	Relative Enzyme Activity (%) (1 mM)	Relative Enzyme Activity (%) (10 mM)
Blank	100 ± 0.8	100 ± 2.1
Cu^2+^	99.7 ± 1.3	62.8 ± 3.2
Fe^3+^	90.3 ± 2.3	0.0 ± 0.1
Mg^2+^	94.7 ± 1.7	94.0 ± 1.1
K^+^	94.3 ± 2.9	97.3 ± 0.3
Zn^2+^	93.1 ± 2.1	77.3 ± 1.5
Ni^2+^	87.3 ± 1.5	86.5 ± 0.8
Co^2+^	99.2 ± 3.1	95.6 ± 1.4
Mn^2+^	110.3 ± 1.2	115.4 ± 2.7
Al^3+^	91.3 ± 0.6	0.6 ± 0.2
Ca^2+^	97.1 ± 1.4	98.1 ± 1.9

**Table 5:** Effect of protease inhibitors, denaturants, and surfactants on the activity of the G4 protease.

Inhibitor/Denaturing/Surfactant Agent	Residual Activity (%)	
None	100 ± 0.7	100 ± 1.1
Inhibitor/Denaturing agents	1 mmol/L	10 mmol/L
PMSF	20.1 ± 1.2	2.7 ± 0.4
EDTA	62.5 ± 0.6	48.9 ± 1.2
1, 10-Phenanthroline	99.0 ± 1.7	98.5 ± 1.0
Dithiothreitol	92.3 ± 1.3	90.5 ± 1.5
		
Surfactant agents	1% (*v*/*v*)	10% (*v*/*v*)
Tween 80	102.5 ± 0.9	105.6 ± 0.5
OP-10	94.6 ± 2.1	101.3 ± 1.7

## Data Availability

The data presented in this study are available on request from the corresponding author or the first author.
